# Manipulation of Insect Vectors’ Host Selection Behavior by Barley Yellow Dwarf Virus Is Dependent on the Host Plant Species and Viral Co-Infection

**DOI:** 10.3390/life12050644

**Published:** 2022-04-26

**Authors:** Nami Minato, Shuichi Hatori, Azusa Okawa, Kai Nakagawa, Mantaro Hironaka

**Affiliations:** 1 Institute of Science and Technology, Niigata University, Niigata 950-2181, Japan; 2 Graduate School of Science and Technology, Niigata University, Niigata 950-2181, Japan; f21d058b@mail.cc.niigata-u.ac.jp (S.H.); f22d050a@mail.cc.niigata-u.ac.jp (K.N.); 3 Graduate School of Agriculture, Tokyo University of Agriculture and Technology (TUAT), Tokyo 183-8509, Japan; s215733x@st.go.tuat.ac.jp; 4 Faculty of Bioresources and Environmental Sciences, Ishikawa Prefectural University, Nonoichi 921-8836, Japan; hironaka@ishikawa-pu.ac.jp

**Keywords:** plant virus, insect vector, co-infection, host selection behavior, *Brachypodium distachyon*, aphid

## Abstract

Previous studies have shown that vector-borne viruses can manipulate the host selection behavior of insect vectors, yet the tripartite interactions of pathogens, host plants and insect vectors have been documented only in a limited number of pathosystems. Here, we report that the host selection behavior of the insect vector of barley yellow dwarf virus-PAV (BYDV-PAV) and cereal yellow dwarf virus-RPS (CYDV-RPS) is dependent on the host plant species and viral co-infection. This study shows that a model cereal plant, *Brachypodium distachyon,* is a suitable host plant for examining tripartite interactions with BYDV-PAV and CYDV-RPS. We reveal that BYDV-PAV has a different effect on the host selection behavior of its insect vector depending on the host plant species. Viruliferous aphids significantly prefer non-infected plants to virus-infected wheat plants, whereas viral infection on a novel host plant, *B. distachyon*, is not implicated in the attraction of either viruliferous or nonviruliferous aphids. Furthermore, our findings show that multiple virus infections of wheat with BYDV-PAV and CYDV-RPS alter the preference of their vector aphid. This result indicates that BYDV-PAV acquisition alters the insect vector’s host selection, thereby varying the spread of multiple viruses.

## 1. Introduction

The majority of plant viruses are transmitted by vector insects [1]. In recent years, increased attention has focused on the tripartite interactions among plants, insect-borne viruses, and insect vectors. An extensive literature documents that plant viruses change host plant phenotypes that have implications for viral transmission. Some insect-borne viruses can manipulate the host selection behavior of vector insects [2,3,4]; however, the tripartite interaction among pathogens, host plants and insect vectors has been documented only in a limited number of pathosystems [5]. In some persistent viruses, nonviruliferous insect vectors preferentially respond to virus-infected plants compared to non-infected plants, whereas viruliferous vectors are attracted to non-infected plants [6,7,8]. A few studies have disclosed the mechanisms underlying viral manipulation of insect vector behavior through volatiles and nutrients [8,9,10,11]; however, the complete picture of how altered host selection behavior is accomplished remains largely unknown.

Yellow dwarf virus species (YDVs) cause one of the most economically important diseases of cereal crops worldwide. The causal agents, barley yellow dwarf virus (BYDV) and cereal yellow dwarf virus (CYDV), are endemic to all continents except Antarctica and infect Poaceae plants, such as wheat, barley, oats, rye and rice [12,13]. In addition, BYDVs’ infection of some dicotyledonous weeds was also recently reported [14]. These viruses induce leaf yellowing and reddening on host plants, reduce crop quality and yields [12,15], and are mentioned as runners-up among the top 10 most important plant viruses worldwide [16,17]. In Japan, two species of YDVs, barley yellow dwarf virus-PAV (BYDV-PAV; family *Luteoviridae*, genus *Luteovirus*) and cereal yellow dwarf virus-RPS (CYDV-RPS; family *Luteoviridae*, genus *Polerovirus*) are reported to be the causal pathogens of yellow dwarf disease on cereals and Poaceae weeds [18,19,20,21]. YDVs are transmitted by aphids in a persistent manner with a latent period, however, they are not transmitted vertically in plants or insects. Among BYDVs’ interactions with crop hosts, insect vectors and the virus, maturing plants are the “dead-end” hosts for BYDVs that must be vectored to green host plants [22]. The evolution of BYDV-modified vector behavior and/or host attractiveness has been implicated in studies using a spatially explicit computer simulation [23]. Indeed, several studies have reported BYDVs’ contribution to altering plant phenotypes for enhancing vector attractiveness and palatability [11,24,25,26].

Plants frequently interact with multiple viruses simultaneously. Among YDVs, BYDVs and CYDVs frequently co-infect host plants in fields around the world, although the most common set of virus species in mixed infections depends on the country/region [27,28,29,30]. In general, co-infection induces more severe symptoms in host plants. For instance, co-infection with BYDV-PAV and CYDV-RPS causes the annual crop *Avena sativa* (oats) to be more stunted compared to a single infection of each viral species [31]. Furthermore, co-infection with BYDV-PAV severely reduces the biomass of host plants [30]. Although little is known about the effects of virus co-infection in manipulating insect vector behavior for host plant selection, limited evidence indicates that mixed infections could significantly influence host-vector interactions [32,33,34].

To address how host plant species affect the host selection behavior of viral vector insects, we identified *Brachypodium distachyon* as a novel host plant for BYDV-PAV and CYDV-RPS. To explore the effects of BYDV and CYDV on the host selection behavior of its insect vector depending on multiple host plant species, we implemented dual-choice bioassays to assess the contribution of BYDV-PAV to the host preference of bird cherry-oat aphids, *Rhopalosipum padi* (Hemiptera: Aphididae). We found that viruliferous aphids significantly preferred non-infected wheat plants to plants infected with a single virus. In contrast, virus infection of *B. distachyon*, a novel host plant, was not implicated in attracting viruliferous or nonviruliferous aphids. Furthermore, wheat infected with more than one virus, i.e., BYDV-PAV and CYDV-RPS, attracted more viruliferous aphids harboring BYDV-PAV than wheat infected with BYDV alone. This result illustrates that BYDV-PAV acquisition alters the host selection of its insect vector to spread multiple viruses.

## 2. Materials and Methods

### 2.1. Plant Materials and Growth Conditions

*Brachypodium distachyon* Bd21 plants (psb00001) were planted and grown in a plant growth chamber with a 20 h light/4 h dark photoperiod at 24 °C. Common wheat (*Triticum aestivum* cv. Norin 61) plants were grown in a plant growth chamber with a 16 h light/8 h dark photoperiod at 22 °C. During the light periods, the light intensity was 120 µmolm^−2^s^−1^.

### 2.2. Insect Colonies and Virus Inoculation

A colony of nonviruliferous bird cherry-oat aphids (*Rhopalosipum padi*) was reared on barley (*Hordeum vulgare* cv. Minori-mugi) plants growing in a plant growth chamber with a 16 h light/8 h dark photoperiod at 22 °C and a light intensity of 120 µmolm^−2^s^−1^. A viruliferous colony of aphids was maintained on BYDV- or CYDV-infected barley plants under the same environmental conditions as the nonviruliferous colony.

Isolates of BYDV-PAV and CYDV-RPS, maintained by the mass transfer of *R. padi* on barley, were used for the virus inoculation. To inoculate 3-week or 30-day old *B. distachyon* and 2-week old common wheat with BYDV-PAV and/or CYDV-RPS, plants covered with plastic and mesh cages were infested with viruliferous aphids. For the preference tests, aphids were removed from the inoculated plants after 3 days. For the sham inoculations, plants were infested with the nonviruliferous *R. padi.* Virus infection was confirmed in all inoculated plants by reverse transcription-polymerase chain reaction (RT-PCR) detection of the BYDV-PAV and/or CYDV-RPS RNA with the PrimeScript One-Step RT-PCR Kit, Ver. 2 (Dye Plus) (TaKaRa Bio Inc., Shiga, Japan). 

### 2.3. Gene Expression Analysis

Reverse transcription-quantitative PCR (RT-qPCR) assays were used to determine the relative transcript levels of selected genes. Assays were performed as described previously [35] with specific primers (Supplementary Table S1), PrimeScript RT Master Mix (Perfect Real Time), and TB Green *Premix Ex Taq* II (TaKaRa Bio Inc.) on a Thermal Cycler Dice Real-Time System III (TP970; TaKaRa Bio Inc.). Fold changes were calculated using the expression of a housekeeping gene *BdUbi4* (Bradi3g04730) as the internal control. Three to four biological replicates were used for each experiment. The *BdUbi4* gene was amplified using primers that were described previously [36]. 

### 2.4. Aphid Preference Tests

Insect dual-choice experiments were conducted in two types of arenas: one type provided cues for host selection throughout the test period (Arena TT), and the other type had fewer cues for sensing other plants after first arrival (Arena Y). Arena TT, adapted from Ingwell et al. [6], allowed aphids to settle on, feed and move between two leaves for each pair-wise test. For each replicate, 50 individual apterous aphids were introduced from the vial to the arena through the tube and monitored every 12 h for 72 h (switching from darkness to white light every 12 h). Each test was replicated three times (in total, 150 viruliferous/nonviruliferous aphids). Arena Y was in a platform consisting of a glass Y-shaped tube (15 mm inner diameter; 100 mm arm length; Figure S1). Since the two arms of the Y-tube were directly connected to the plastic cage covering the plants, aphids were able to be in direct contact with the treated leaves that were inserted 20 mm into each arm of the Y-shaped tube. In Arena Y, the host plant choice was registered when an aphid stayed in one of the arms after 20 min or after 12 h from the introduction in the white light condition. For the 20 min test, a single aphid was released from a point 50 mm away from the central tube. In the 12 h experiment, a population of 10 individuals was introduced. Each test was replicated three times. A total of 150 viruliferous and nonviruliferous aphids were assessed in the 12 h experiment, and a total of 90 aphids were tested in the 20 min test. 

### 2.5. Data Analysis

Statistical analyses were conducted using R (ver. 3.5.2) with R studio (ver. 1.1.463; RStudio, PBC., Boston, MA, USA). Data for plant heights were analyzed using the Student’s *t*-test and the Tukey–Kramer test. A comparison of the viral accumulation was analyzed using the Steel–Dwass test and the Wilcoxon rank sum test. For the insect dual-choice bioassays, data were analyzed using a binomial test for identifying the preference and *χ*^2^ tests for detecting differences in preference between viruliferous and nonviruliferous aphids.

## 3. Results

### 3.1. BYDV-PAV and CYDV-RPS Infect Brachypodium Distachyon via an Aphid Vector

**Figure 1:**
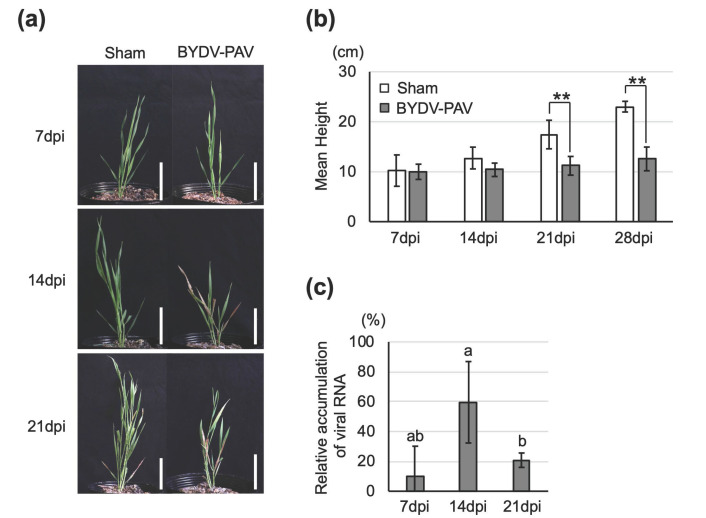
BYDV-PAV infects *Brachypodium distachyon*. (**a**) Symptom development of BYDV-PAV-infected *B. distachyon*. Three-week-old *B. distachyon* plants were sham-inoculated with nonviruliferous aphids or inoculated with BYDV-PAV viruliferous aphids. Plants were photographed 7, 14, and 21 days post-inoculation (dpi). Scale bars represent 5 cm. (**b**) Growth rate of BYDV-PAV-infected *B. distachyon* plants compared to sham-inoculated plants at 1 to 4 weeks post-inoculation. White bars represent the mean plant height of sham-inoculated plants; dark gray bars represent the mean plant height of BYDV-PAV infected plants. Bars represent the means ± SD. (n = 5). Asterisks indicate significant differences (Student’s *t*-test, ** *p* < 0.01). (**c**) The accumulation levels of BYDV-PAV RNA in plants single-infected with BYDV-PAV. Lowercase letters indicate significant differences as determined by the Steel–Dwass test (*p* < 0.05).

To evaluate if yellow dwarf viruses (YDVs) widely infect *Brachypodium distachyon*, we assessed the symptoms and virus titer of single- and co-infected plants. First, barley yellow dwarf virus-PAV (BYDV-PAV) was inoculated on *B. distachyon* with the aphid vector *Rhopalosiphum padi*. Over 88% of inoculated plants became infected at 7 days post-inoculation (dpi). BYDV-PAV single-infected *B. distachyon* exhibited leaf reddening after 14 dpi and significant dwarfing at 21–28 dpi (Figure 1a,b; Student’s *t*-test, 21 dpi: *t* = 4.096, *p* = 0.003; 28 dpi: *t* = 8.969, *p* < 0.001). These observations were consistent with previous observations of BYDV-GAV-infected *B. distachyon* [37]. Reverse transcription-quantitative PCR (RT-qPCR) of virus-infected *B. distachyon* plants revealed that the accumulation of viral RNA was significantly greater at 14 dpi than at 21dpi (Figure 1c; Steel–Dwass test, 7–14 dpi: *p* = 0.122; 7–21 dpi: *p* = 0.026; 14–21 dpi: *p* = 0.038).

**Figure 2:**
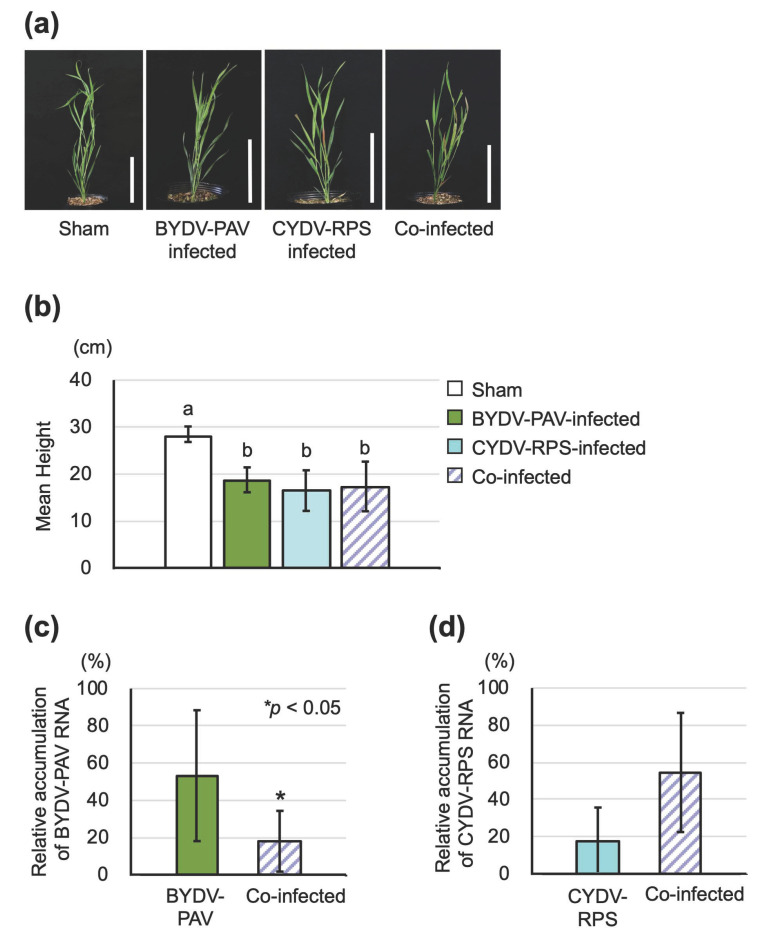
BYDV-PAV and CYDV-RPS co-infection of *Brachypodium distachyon*. (**a**) Comparison of symptom development after single- or co-infection of *B. distachyon* with BYDV-PAV and/or CYDV-RPS. *B. distachyon* plants (30-days old) were inoculated and photographed 21-days post-inoculation (dpi). Scale bars represent 10 cm. (**b**) Growth rate of BYDV-PAV- and/or CYDV-RPS-infected *B. distachyon* plants compared to sham-inoculated plants at 21 dpi. Bars represent the means ± SD. (n = 4). Lowercase letters indicate significant differences (Tukey–Kramer test, *p* < 0.05). (**c**) The accumulation levels of BYDV-PAV RNA in plants at 7 dpi after single-infection with BYDV-PAV or co-infection. Bars represent the means ± SD. (n = 4). Asterisks indicate significant differences by the Wilcoxon rank sum test (* *p* < 0.05). (**d**) The accumulation levels of CYDV-RPS RNA in plants at 7 dpi after single-infection with CYDV-RPS or co-infection. Bars represent the means ± SD. (n = 3). (**b**–**d**) Colored bars indicate each type of infection treatment: sham-inoculated (white), BYDV-PAV (green), CYDV-RPS (blue), BYDV-PAV + CYDV-RPS co-infection (purple hatched).

Co-infected *B. distachyon* with BYDV-PAV and cereal yellow dwarf virus-RPS (CYDV-RPS) showed leaf reddening, similar to that of BYDV-PAV-infected and CYDV-RPS single-infected plants (Figure 2a). Plant heights of co-infected plants were significantly shorter than sham-inoculated plants; however, plant heights were not significantly different from those of single-infected plants (Figure 2b; Tukey–Kramer test, sham-BYDV: *p* = 0.024; sham-CYDV: *p* = 0.011; sham-co-infection: *p* = 0.010; BYDV-CYDV: *p* = 0.965; BYDV- co-infection: *p* = 0.951; CYDV-co-infection: *p* = 1.000). Our results suggest that co-infection of BYDV-PAV and CYDV-RPS isolated from Japan does not cause more significant damage to *B. distachyon* plants than single-infection with these virus species. Virus accumulation of BYDV-PAV was greater in single-infected *B. distachyon* plants than in co-infected plants at 7 dpi (Figure 2c; Wilcoxon rank sum test, *p* = 0.029). In contrast, the transcript abundance of CYDV-RPS was not significantly different between single-infected and co-infected plants at 7 dpi (Figure 2d).

### 3.2. BYDV-PAV Affects the Host Preference of the Aphid Vector R. padi on Wheat

**Figure 3:**
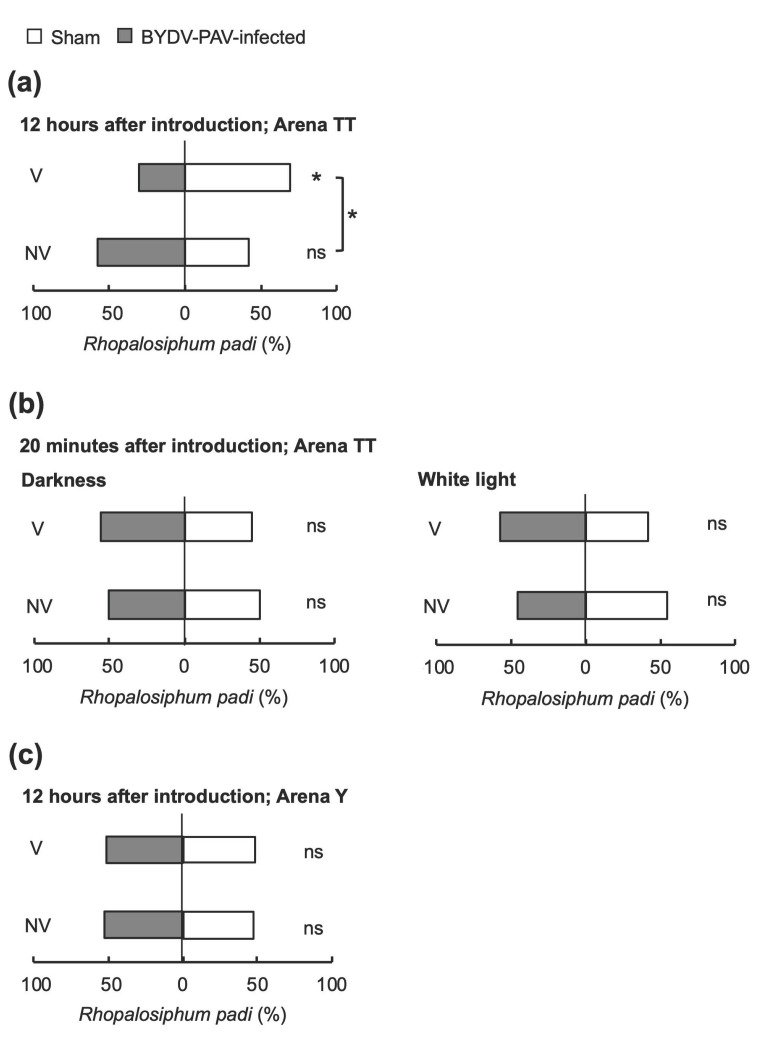
Viruliferous aphids *Rhopalosiphum padi* harboring BYDV-PAV demonstrate a preference for non-infected wheat plants. (**a**) Mean proportion of viruliferous (V) and nonviruliferous (NV) apterous aphids responding to wheat in a dual-choice Arena TT at 12 h after introduction; (**b**) Mean proportion of viruliferous (V) and nonviruliferous (NV) aphids responding to wheat in a dual-choice Arena TT 20 min after introduction under darkness or white light. (**c**) Mean proportion of viruliferous (V) and nonviruliferous (NV) aphids responding to wheat in a dual-choice Arena Y 12 h after introduction. The graphs represent the percentage of vector aphid *R. padi* adults and nymphs found on each test plant within a single-choice cage. Sham-inoculated wheat plants were tested in comparison with BYDV-PAV-infected plants at 14 dpi. The asterisk indicates a significant preference within a dual-choice test: *ns*, not significant; asterisk, *p* < 0.05 (binomial test).

Dual-choice bioassays were implemented to assess whether the Japanese isolate of BYDV-PAV influences the preference of its vector aphid, *R. padi*. We conducted pair-wise preference tests that allowed visual, taste, volatile and contact cues throughout the test period separately for viruliferous and nonviruliferous apterous aphids. When aphids were able to exploit cues from other host candidates after arrival to the choice of plant in Arena TT, a 12 h or longer period resulted in viruliferous apterous *R. padi* preferring non-infected wheat plants to virus-infected plants, whereas the attractiveness of infected plants to nonviruliferous aphids was not significant (Figure 3a and Figure S2; binomial test, V: *p* = 0.005; NV: *p* = 0.473; *χ*^2^ test, *χ*^2^ = 5.205, *p* = 0.023). However, in a short twenty-minute observation in Arena TT, both nonviruliferous and viruliferous aphids did not show a preference between non-infected wheat plants and infected plants, either under darkness or white light (Figure 3b; binomial test, V_dark_: *p* = 1.000; NV_dark_: *p* = 1.000; Fisher’s exact test (darkness), *p* = 1.000; V_light_: *p* = 0.557; NV_light_: *p* = 0.832; *χ*^2^ test (white light), *χ*^2^ _light_= 0.309, *p* = 0.578), suggesting that the selection period has an important role in the aphids’ host selection behavior.

To explore crucial cues for the host selection behavior of insect vectors, we performed further pair-wise preference tests using Arena Y. With Arena Y, both viruliferous and nonviruliferous aphids exhibited no preference between the virus-infected and non-infected plants, either after 20 min (Figure S3) or 12 h (Figure 3c; binomial test, V: *p* = 0.916; NV: *p* = 0.741; *χ*^2^ test, *χ*^2^ < 0.001, *p* = 0.983). These results suggest that opportunities for sensing stimuli after the first arrival, influence the host selection behavior of insect vectors more than the selection time.

### 3.3. Alteration of Aphids’ Host Preference by BYDV-PAV Depends on the Host Poaceae Species

**Figure 4:**
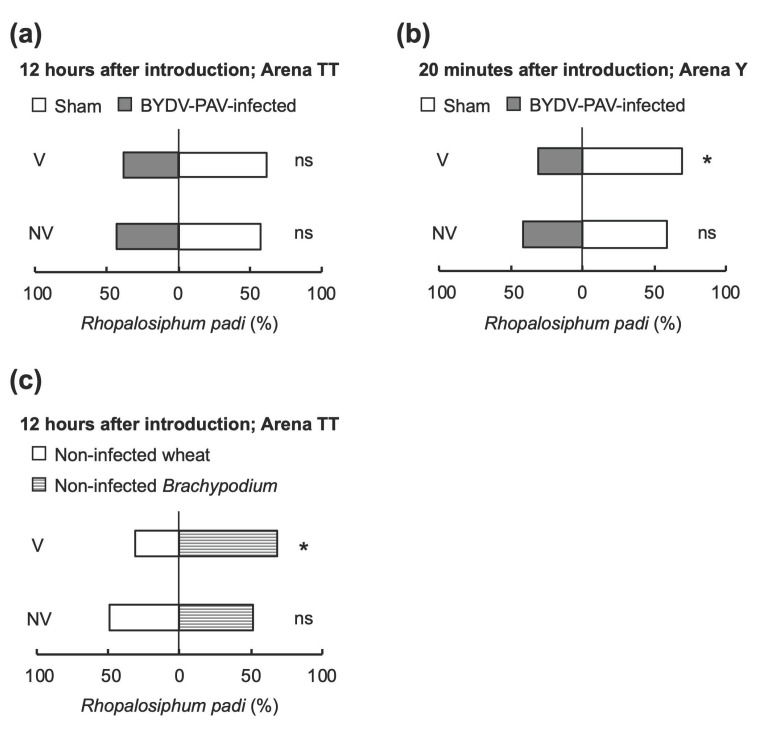
The attraction of vector aphids *Rhopalosiphum padi* to BYDV-PAV-infected and non-infected *Brachypodium distachyon* does not differ. (**a**) Mean proportion of viruliferous (V) and nonviruliferous (NV) apterous aphids responding to *Brachypodium* 12 h after introduction in a dual-choice Arena TT. (**b**) Mean proportion of viruliferous (V) and nonviruliferous (NV) aphids responding to *Brachypodium* 20 min after introduction to a dual-choice Arena Y. (**c**) Mean proportion of viruliferous (V) and nonviruliferous (NV) aphids responding to non-infected wheat and non-infected *Brachypodium* plants 12 h after introduction to a dual-choice Arena TT. The graphs represent the percentage of *R. padi* adults and nymphs found on each test plant within a single-choice cage. (**a**,**b**) Sham-inoculated *B. distachyon* plants were tested compared to BYDV-PAV-infected plants at 14 dpi. Asterisks indicate a significant preference within a dual-choice test: *ns*, not significant; asterisk, *p* < 0.05 (binomial test).

To investigate further, the effect of the host plant species on host selection by insect vectors, we compared the aphid behavior in response to the BYDV-PAV-infected *B. distachyon* plants to those in response to the sham-inoculated plants. In Arena TT with *B. distachyon*, virus infection was not implicated in the attraction of viruliferous nor nonviruliferous aphids by 12 h after introduction (Figure 4a; binomial test, V: *p* = 0.068; NV: *p* = 0.321; *χ*^2^ test, *χ*^2^ = 0.153, *p* = 0.695). Viruliferous aphids rarely preferred non-infected plants during the 72 h test period (Figure S4). In Arena Y, the attractiveness of non-infected plants for viruliferous aphids was slightly greater than that of virus-infected plants by 20 min after introduction (Figure 4b; binomial test, V: *p* = 0.002; NV: *p* = 0.188; *χ*^2^ test, *χ*^2^ = 1.763, *p* = 0.414). However, the short observation period caused heterogeneity in aphids’ preference, indicating that viruliferous aphids do not exhibit a stable preference for non-infected plants in *Brachypodium*, even when they have sufficient opportunities for sensing stimuli and an extended selection time.

In consideration that viruliferous aphids significantly preferred non-infected wheat in Arena TT (Figure 3), we hypothesized that the preference of *R. padi* aphids might be affected only by their preferred host plant species, even though they can transmit the virus to wheat and *Brachypodium*. To address the preference of aphids responding to plant species as their feeding host, we studied the host selection behavior of viruliferous and nonviruliferous *R. padi* against non-infected wheat and non-infected *B. distachyon*. Viruliferous aphids tended to prefer *Brachypodium*, and nonviruliferous aphids exhibited no preference between wheat and *B. distachyon* as their host plants (Figure 4c; binomial test, V: *p* = 0.016; NV: *p* = 1.000; *χ^2^* test, *χ^2^* = 2.269, *p* = 0.132). Since the preference of viruliferous aphids differed between the two host plant species, wheat and *B. distachyon*, it seems clear that host selection behavior is not fully determined by vector manipulation. It is equally evident that insect vectors can distinguish phenotypes of infected and non-infected plants as key cues only on some plant species. These findings indicate that opportunities for sensing stimuli are crucial for the host selection behavior of aphids, yet the host plant species has a stronger effect.

### 3.4. Insect Vectors Harboring Only BYDV-PAV Facilitated the Co-Infection of Multiple Viruses on Wheat

**Figure 5:**
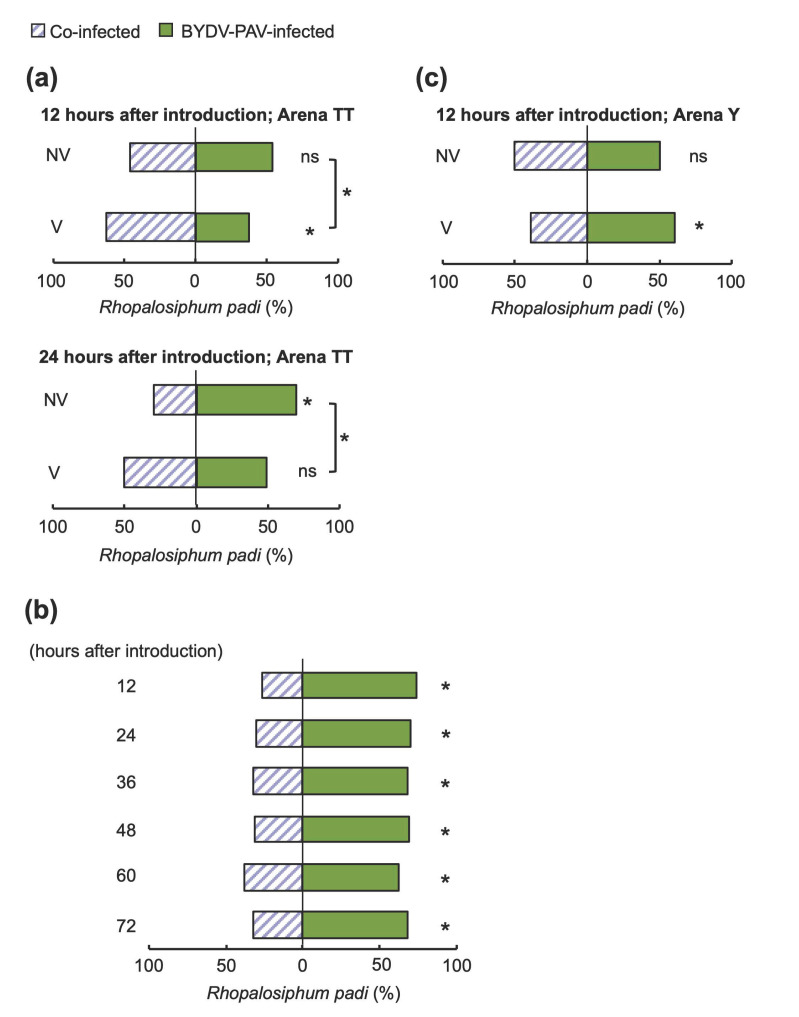
Viruliferous aphids harboring BYDV-PAV preferred virus co-infected wheat plants to single-infected plants. (**a**) Mean proportion of nonviruliferous (NV) and viruliferous (V) apterous aphids responding to wheat 12 and 24 h after introduction to a dual-choice Arena TT. Aphid preferences significantly different between single- and co-infected plants are indicated with asterisks between the bars of NV and V (*χ*^2^ test, * *p* < 0.05). (**b**) The mean proportion of nonviruliferous aphids responding to wheat over 12 to 72 h after introduction into a dual-choice Arena TT. (**c**) The mean proportion of nonviruliferous (NV) and viruliferous (V) aphids responding to wheat 12 h after introduction to a dual-choice Arena Y. The graphs represent the percentage of vector aphid *Rhopalosiphum padi* adults and nymphs found on each test plant within a single-choice cage. BYDV-PAV single-infected wheat plants were tested compared to BYDV-PAV and CYDV-RPS co-infected plants at 14 dpi. An asterisk indicates a significant preference within a dual-choice test: *ns*, not significant; asterisk, *p* < 0.05 (binomial test).

The effects of co-infection by multiple plant viruses on tripartite interactions are largely unexplored. Here, we investigated the host preference of the vector aphid *R. padi* for plants co-infected with BYDV-PAV and CYDV-RPS. We conducted pair-wise preference tests for viruliferous and nonviruliferous apterous aphids. In Arena TT, which allowed aphids to sense cues for host selection throughout the test period, BYDV-PAV viruliferous and nonviruliferous aphids exhibited a different preference between BYDV-PAV single-infected and co-infected plants 12 h after insect introduction (Figure 5a; *χ*^2^ test, *χ*^2^ = 4.31, *p* = 0.038). Aphid preference was influenced by the virus co-infecting the wheat plants: viruliferous aphids tended to prefer co-infected plants (Figure 5a; binomial test, NV: *p* = 0.572; V: *p* = 0.012). By 24 h after introduction, nonviruliferous aphids significantly preferred BYDV-PAV single-infected plants to co-infected plants (Figure 5a; binomial test, NV: *p* = 0.0002; V: *p* = 1.000; *χ*^2^ test, *χ*^2^ = 7.838, *p* = 0.005). The preference for nonviruliferous aphids over single-infected plants was maintained throughout the 72 h test (Figure 5b; binomial test, *p* < 0.001 for each period). In the choice test using Arena Y, which provides fewer cues for sensing other plants after introduction, the attraction of single-infected and co-infected plants did not significantly differ between viruliferous and nonviruliferous aphids 12 h after insect introduction (Figure 5c; binomial test, NV: *p* = 1.000; V: *p* = 0.024; *χ*^2^ test, *χ*^2^ = 2.567, *p* = 0.109). These results indicate that multiple virus infections with BYDV-PAV and CYDV-RPS alter the host selection behavior of their vector aphid.

## 4. Discussion

For studying insect-borne pathogens, an experimental system needs to be established that includes host plants, both the pathogen and the vector insect. This study provides clear evidence that *Brachypodium distachyon*, which has a completely sequenced genome and a shorter life cycle than wheat [38,39], is a suitable host plant for examining tripartite interactions among plants, insect-borne pathogens, and insect vectors. BYDV-PAV and CYDV-RPS could (co-)infect *B. distachyon*, and BYDV-PAV infected *B. distachyon* plants with clearer symptoms than those found on infected wheat (Figure 1 and Figure 2). We also documented that the Japanese isolate of BYDV-PAV had a different effect on the host selection behavior of its insect vector between two host plant species. Viruliferous aphids significantly preferred non-infected plants to virus-infected wheat plants (Figure 3). In contrast, virus infection of a novel host plant *B. distachyon* was not implicated in the attraction of either viruliferous or nonviruliferous aphids (Figure 4). Furthermore, our findings highlight that BYDV-PAV acquisition alters the host selection of its insect vector for spreading multiple viruses (Figure 5).

Ingwell et al. [6] proposed a “Vector Manipulation Hypothesis” (VMH) that posits the direct manipulation of insect vectors on host selection behavior by plant pathogens. This theory is in addition to the classic “Host Manipulation Hypothesis” (HMH) that proposes that pathogens change host behavior to enhance transmission. We found that the two host plant species, wheat and *B. distachyon*, differed in the preference of viruliferous aphids (Figure 4), indicating that their host selection behavior is not entirely determined by vector manipulation, but that host plant manipulation is needed. Our findings support the thesis that opportunities for sensing stimuli are crucial for the host selection behavior of aphids, yet the host plant species has a stronger effect.

In the longer observation periods of 12 to 72 h, light conditions did not influence the preference of aphids to select wheat and *B. distachyon*. This finding suggests that visual cues scarcely play a role in host selection behavior. Although YDVs appeared to not require lights for vector manipulation, another group of insect-borne plant viruses, begomovirus, utilize red light as an environmental factor to alter the behavior of its whitefly vector [40]. Thus, insect-borne viruses might have evolved diverse strategies for manipulating insect vectors. In the Arena Y system with the cereal model plant *B. distachyon*, the attraction of non-infected plants for viruliferous aphids was slightly greater than that of virus-infected plants (Figure 4b). However, the short observation period caused heterogeneity in aphids’ preference, suggesting that the selection period has an important role in the host selection behavior of aphids. The virus may have compelling impacts on the initiation and duration of vector feeding behavior and vector attraction mediated by odor or visual cues [26].

Our results revealed that multiple virus infections with BYDV-PAV and CYDV-RPS alter the host selection behavior of the insect vector (Figure 5), a result consistent with two studies of other insect-borne viruses [33,41]. These data suggest that BYDV-PAV acquisition likely alters the host selection of its insect vector to spread multiple viruses. Wang et al. [32] reported that brown planthoppers (BPHs; *Nilaparvata lugens*) preferred non-virus infected rice plants before virus acquisition, whereas rice ragged stunt virus (RRSV)-carrying BPHs preferred Southern rice black-streaked dwarf virus (SRBSDV)-infected rice plants. These findings indicate that plant viruses might alter the host selection preference of insect vectors to enhance their spread. In this case, RRSV and SRBSDV did not share their insect vectors: RRSV is transmitted by BPHs, whereas another insect species, the white-backed planthopper (WBPH; *Sogatella furcifera*), transfers SRBSDV. Since the vector aphid of BYDV-PAV, *R. padi*, can also transmit CYDV-RPS, manipulating the vector’s host selection behavior by one virus is likely to contribute more directly to the spread of another virus.

This study provides that BYDV-PAV single-infected *B. distachyon* plants accumulate higher viral loads compared to co-infected plants during the early stages of infection, whereas the CYDV titer does not differ between single- and co-infected host plants (Figure 2). In addition, the BYDV titer dynamics were observed during the single-infection period (Figure 1). Some previous studies reported that low virus titers lead to limited transmission, and high titers enhance the transmission by influencing the performance of aphid vectors via odor cues and plant defense during single infections [24,42]. Our preliminary experiments show that levels of BYDV-PAV and CYDV-RPS accumulation were synergistically increased by co-infection of wheat at the late stages of infection. Therefore, additional work will be needed to confirm the contribution of virus titer to the host selection behavior of the insect vector when co-infection has occurred.

## Supplementary Materials

The following supporting information can be downloaded at: https://www.mdpi.com/article/10.3390/life12050644/s1, Figure S1: The experimental setup of the dual-choice test, Arena Y; Figure S2: The preference of viruliferous aphids *Rhopalosiphum padi* harboring BYDV-PAV for non-infected wheat plants is maintained throughout the 72 h test; Figure S3: Viruliferous and nonviruliferous aphids exhibit no preference between virus-infected and non-infected wheat plants after 20 min; Figure S4: Viruliferous aphids harboring BYDV-PAV do not exhibit a stable preference for non-infected *Brachypodium distachyon* plants throughout the 72 h test; Table S1: Primers used for RT-qPCR analysis.

## References

[1] Hogenhout S.A., Ammar E.-D., Whitfield A.E., Redinbaugh M.G. (2008). Insect vector interactions with persistently transmitted viruses. Annu. Rev. Phytopathol..

[2] Mauck K., Bosque-Pérez N.A., Eigenbrode S.D., De Moraes C.M., Mescher M.C. (2012). Transmission mechanisms shape pathogen effects on host–vector interactions: Evidence from plant viruses. Funct. Ecol..

[3] Eigenbrode S.D., Bosque-Pérez N.A., Davis T.S. (2018). Insect-borne plant pathogens and their vectors: Ecology, evolution, and complex interactions. Annu. Rev. Entomol..

[4] Mauck K.E., Chesnais Q., Shapiro L.R. (2018). Evolutionary determinants of host and vector manipulation by plant viruses. Adv. Virus Res..

[5] Mauck K.E., Chesnais Q. (2020). A synthesis of virus-vector associations reveals important deficiencies in studies on host and vector manipulation by plant viruses. Virus Res..

[6] Ingwell L.L., Eigenbrode S.D., Bosque-Pérez N.A. (2012). Plant viruses alter insect behavior to enhance their spread. Sci. Rep..

[7] Rajabaskar D., Bosque-Pérez N.A., Eigenbrode S.D. (2014). Preference by a virus vector for infected plants is reversed after virus acquisition. Virus Res..

[8] Mauck K.E. (2016). Variation in virus effects on host plant phenotypes and insect vector behavior: What can it teach us about virus evolution?. Curr. Opin. Virol..

[9] Mauck K.E., De Moraes C.M., Mescher M.C. (2016). Effects of pathogens on sensory-mediated interactions between plants and insect vectors. Curr. Opin. Plant Biol..

[10] Ziegler-Graff V. (2020). Molecular insights into host and vector manipulation by plant viruses. Viruses.

[11] Porras M.F., Navas C.A., Marden J.H., Mescher M.C., De Moraes C.M., Pincebourde S., Sandoval-Mojica A., Raygoza-Garay J.A., Holguin G.A., Rajotte E.G. (2020). Enhanced heat tolerance of viral-infected aphids leads to niche expansion and reduced interspecific competition. Nat. Commun..

[12] Burnett P.A. (1990). World Perspectives on Barley Yellow Dwarf: Proceedings of the International Workshop July 6–11, 1987, Undine Italy.

[13] İLbaĞI H., Aydin Ö. (2020). Firs t Report of Yellow Dwarf Viruses (YDVs) in the Rice Fields in the Trakya Region of Turkey. Ekin J. Crop Breed. Genet..

[14] Ilbaği H., Çitir A., Kara A., Uysal M., Olden F.A. (2019). First report of Barley Yellow Dwarf Viruses (BYDVs) on dicotyledonous weed hosts in Turkey. Cereal Res. Commun..

[15] Riedell W.E., Kieckhefer R.W., Langham M.A.C., Hesler L.S. (2003). Root and shoot responses to bird cherry-oat aphids and barley yellow dwarf virus in spring wheat. Crop Sci..

[16] Scholthof K.B.G., Adkins S., Czosnek H., Palukaitis P., Jacquot E., Hohn T., Hohn B., Saunders K., Candresse T., Ahlquist P. (2011). Top 10 plant viruses in molecular plant pathology. Mol. Plant Pathol..

[17] Rybicki E.P. (2015). A Top Ten list for economically important plant viruses. Arch. Virol..

[18] Toriyama S., Yora K., Asuyama H. (1968). Occurrence of Barley yellow dwarf virus (BYDV) in Japan. Proceedings of the Autumn Meeting of the Kanto Division, Annual Meeting of the Phytopathological Society of Japan.

[19] Kojima M., Matsubara A., Yanase S., Toriyama S. (1983). The occurrence of barley yellow dwarf disease in Japan. Jpn. J. Phytopathol..

[20] Usugi T., Nakano M., Shinkai A. (1987). Occurrence of barley yellow dwarf virus and wheat yellow leaf virus in Kyushu. Proc. Assoc. Plant Prot. Kyushu.

[21] Fan Y.-J., Namba S., Yamashita S., Doi Y. (1994). Some properties of barley yellow dwarf virus (BYDV) from maize and some cereal plants. Jpn. J. Phytopathol..

[22] Trebicki P. (2020). Climate change and plant virus epidemiology. Virus Res..

[23] McElhany P., Real L.A., Power A.G. (1995). Vector preference and disease dynamics: A study of barley yellow dwarf virus. Ecology.

[24] Jiménez-Martínez E.S., Bosque-Pérez N.A., Berger P.H., Zemetra R.S., Ding H., Eigenbrode S.D. (2004). Volatile cues influence the response of Rhopalosiphum padi (Homoptera: Aphididae) to Barley yellow dwarf virus–infected transgenic and untransformed wheat. Environ. Entomol..

[25] Medina-Ortega K.J., Bosque-Pérez N.A., Ngumbi E., Jiménez-Martínez E.S., Eigenbrode S.D. (2009). Rhopalosiphum padi (Hemiptera: Aphididae) responses to volatile cues from barley yellow dwarf virus–infected wheat. Environ. Entomol..

[26] Porras M., De Moraes C.M., Mescher M.C., Rajotte E.G., Carlo T.A. (2018). A plant virus (BYDV) promotes trophic facilitation in aphids on wheat. Sci. Rep..

[27] Seabloom E.W., Hosseini P.R., Power A.G., Borer E.T. (2009). Diversity and composition of viral communities: Coinfection of barley and cereal yellow dwarf viruses in California grasslands. Am. Nat..

[28] Liu Y., Khine M.O., Zhang P., Fu Y., Wang X. (2020). Incidence and distribution of insect-transmitted cereal viruses in wheat in China from 2007 to 2019. Plant Dis..

[29] Laney A.G., Acosta-Leal R., Rotenberg D. (2018). Optimized yellow dwarf virus multiplex PCR assay reveals a common occurrence of Barley yellow dwarf virus-PAS in Kansas winter wheat. Plant Health Prog..

[30] Mordecai E.A., Hindenlang M., Mitchell C.E. (2015). Differential impacts of virus diversity on biomass production of a native and an exotic grass host. PLoS ONE.

[31] Malmstrom C.M., Bigelow P., Trębicki P., Busch A.K., Friel C., Cole E., Abdel-Azim H., Phillippo C., Alexander H.M. (2017). Crop-associated virus reduces the rooting depth of non-crop perennial native grass more than non-crop-associated virus with known viral suppressor of RNA silencing (VSR). Virus Res..

[32] Wang H., Xu D., Pu L., Zhou G. (2014). Southern rice black-streaked dwarf virus alters insect vectors’ host orientation preferences to enhance spread and increase rice ragged stunt virus co-infection. Phytopathology.

[33] Lightle D., Lee J. (2014). Raspberry viruses affect the behavior and performance of A mphorophora agathonica in single and mixed infections. Entomol. Exp. Appl..

[34] Peñaflor M.F.G.V., Mauck K.E., Alves K.J., De Moraes C.M., Mescher M.C. (2016). Effects of single and mixed infections of Bean pod mottle virus and Soybean mosaic virus on host-plant chemistry and host–vector interactions. Funct. Ecol..

[35] Senshu H., Yamaji Y., Minato N., Shiraishi T., Maejima K., Hashimoto M., Miura C., Neriya Y., Namba S. (2011). A dual strategy for the suppression of host antiviral silencing: Two distinct suppressors for viral replication and viral movement encoded by potato virus M. J. Virol..

[36] Chambers J.P., Behpouri A., Bird A., Ng C.K.Y. (2012). Evaluation of the use of the Polyubiquitin Genes, Ubi4 and Ubi10 as reference genes for expression studies in Brachypodium distachyon. PLoS ONE.

[37] Tao Y., Nadege S.W., Huang C., Zhang P., Song S., Sun L., Wu Y. (2016). Brachypodium distachyon is a suitable host plant for study of Barley yellow dwarf virus. Virus Genes.

[38] Draper J., Mur L.A.J., Jenkins G., Ghosh-Biswas G.C., Bablak P., Hasterok R., Routledge A.P.M. (2001). Brachypodium distachyon. A new model system for functional genomics in grasses. Plant Physiol..

[39] Scholthof K.-B.G., Irigoyen S., Catalan P., Mandadi K.K. (2018). Brachypodium: A monocot grass model genus for plant biology. Plant Cell.

[40] Zhao P., Zhang X., Gong Y., Wang D., Xu D., Wang N., Sun Y., Gao L., Liu S.-S., Deng X.W. (2021). Red-light is an environmental effector for mutualism between begomovirus and its vector whitefly. PLoS Pathog..

[41] Chesnais Q., Couty A., Uzest M., Brault V., Ameline A. (2019). Plant infection by two different viruses induce contrasting changes of vectors fitness and behavior. Insect Sci..

[42] Shi X., Gao Y., Yan S., Tang X., Zhou X., Zhang D., Liu Y. (2016). Aphid performance changes with plant defense mediated by Cucumber mosaic virus titer. Virol. J..

