# Supplementary material for: Manipulation of Insect Vectors’ Host Selection Behavior by Barley Yellow Dwarf Virus Is Dependent on the Host Plant Species and Viral Co-Infection
Source: Life (Basel). 2022 Apr 26;12(5):644. doi: 10.3390/life12050644 (PMC9142937; doi:10.3390/life12050644)
Supplement: Supplementary file 1 [file life-12-00644-s001.zip › life-1665746-supple.pdf]

life

Supplementary Materials

Supplementary Table S1. Primers used for RT-qPCR analysis.

Name	Sequence (5′-3′)	Target	Reference
BdUbi4_F	TGACACCATCGACAACGTGA	*Bradi3g04730* (*Ubi4*)	[Chambers *et al.*, 2012]
BdUbi4_R	GAGGGTGGACTCCTTCTGGA	*Bradi3g04730* (*Ubi4*)	[Chambers *et al.*, 2012]
qBYDV-PAV-RdRp_F	GAATGGTCAGAAGGCCCGAT	BYDV-PAV, RdRp	This study
qBYDV-PAV-RdRp_Rv	GTTGAGCACCAGACCACACT	BYDV-PAV, RdRp	This study
qCYDV-RPS-RdRp_F	AACTGGGAATCAGCGTTCGG	CYDV-RPS, RdRp	This study
qCYDV-RPS-RdRp_Rv	GCGCCAATCTTCATCCTCCA	CYDV-RPS, RdRp	This study

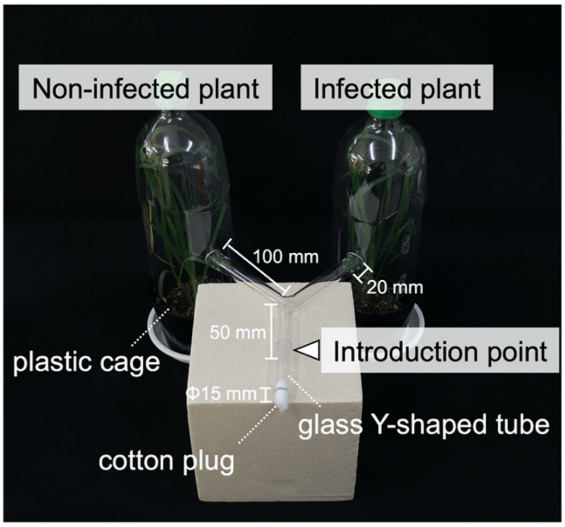

Supplementary Figure S1. The experimental setup of the dual-choice test Arena Y.

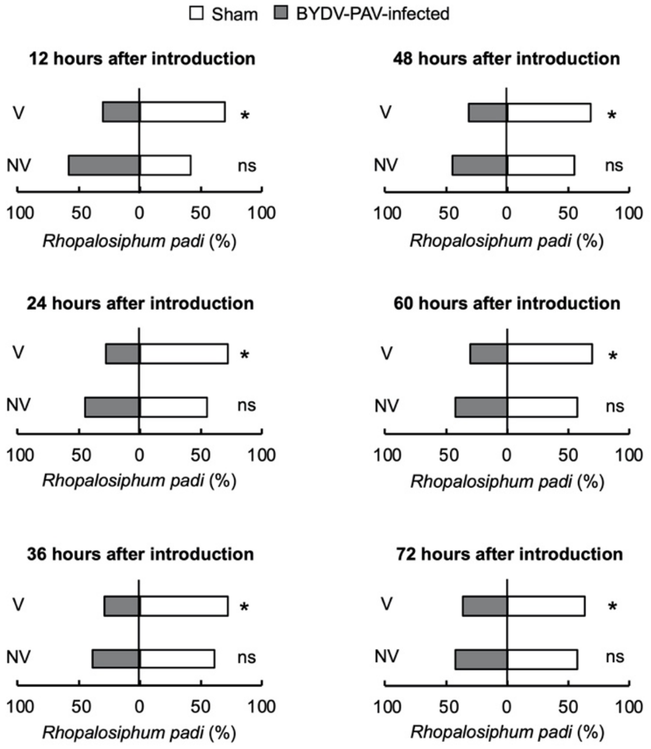

Supplementary Figure S2. The preference of viruliferous aphids *Rhopalosiphum padi* harboring BYDV-PAV for non-infected wheat plants is maintained throughout the 72-hour test. Mean proportion of nonviruliferous (NV) and viruliferous (V) apterous aphids responding to wheat over 12 to 72 hours after introduction into a dual-choice Arena TT. The graphs represent the percentage of vector aphid *R. padi* adults and nymphs found on each test plant within a single-choice cage. Sham-inoculated wheat plants were tested in comparison with BYDV-PAV-infected plants at 14 dpi. The asterisk indicates a significant preference within a dual-choice test: *ns*, not significant; asterisk, $p < 0.05$ (binomial test).

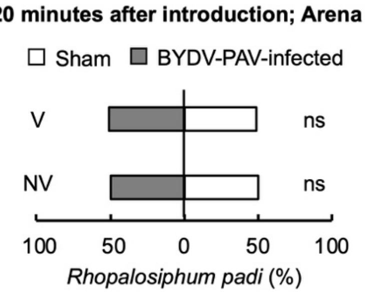

Supplementary Figure S3. Viruliferous and nonviruliferous aphids exhibit no preference between virus-infected and non-infected wheat plants after 20 minutes. Mean proportion of viruliferous (V) and nonviruliferous (NV) aphids responding to wheat in a dual-choice Arena Y 20 minutes after introduction. The graphs represent the percentage of vector aphid *R. padi* adults and nymphs found on each test plant within a single-choice cage. Sham-inoculated wheat plants were tested in comparison with BYDV-PAV-infected plants at 14 dpi. The asterisk indicates a significant preference within a dual-choice test: *ns*, not significant; asterisk, $p < 0.05$ (binomial test).

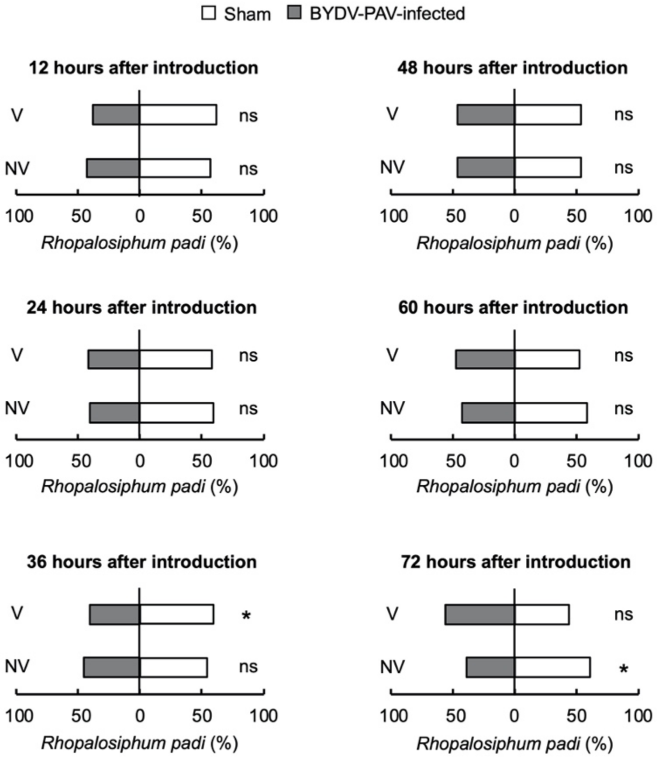

Supplementary Figure S4. Viruliferous aphids harboring BYDV-PAV do not exhibit a stable preference for non-infected *Brachypodium distachyon* plants throughout the 72-hour test. Mean proportion of nonviruliferous (NV) and viruliferous (V) apterous aphids responding to *Brachypodium* plants over 12 to 72 hours after introduction into a dual-choice Arena TT. The graphs represent the percentage of vector aphid *R. padi* adults and nymphs found on each test plant within a single-choice cage. Sham-inoculated *B. distachyon* plants were tested in comparison with BYDV-PAV-infected plants at 14 dpi. The asterisk indicates a significant preference within a dual-choice test: *ns*, not significant; asterisk, $p < 0.05$ (binomial test).
